# Chemokine Fractalkine and Non-Obstructive Coronary Artery Disease—Is There a Link?

**DOI:** 10.3390/ijms25073885

**Published:** 2024-03-30

**Authors:** Aleksandra Stangret, Karol Artur Sadowski, Konrad Jabłoński, Janusz Kochman, Grzegorz Opolski, Marcin Grabowski, Mariusz Tomaniak

**Affiliations:** 1 Chair and Department of Experimental and Clinical Physiology, Laboratory of Centre for Preclinical Research, Medical University of Warsaw, Banacha 1b, 02-097 Warsaw, Poland; aleksandra.stangret@wum.edu.pl; 2 1st Department of Cardiology, Medical University of Warsaw, Banacha 1a, 01-267 Warsaw, Poland; s082685@student.wum.edu.pl (K.A.S.); s080018@student.wum.edu.pl (K.J.); jkochman@wum.edu.pl (J.K.); grzegorz.opolski@wum.edu.pl (G.O.); marcin.grabowski@wum.edu.pl (M.G.)

**Keywords:** fractalkine/CX3CL1, non-obstructive coronary artery disease

## Abstract

Non-obstructive coronary artery disease (NO-CAD) constitutes a heterogeneous group of conditions collectively characterized by less than 50% narrowing in at least one major coronary artery with a fractional flow reserve (FFR) of ≤0.80 observed in coronary angiography. The pathogenesis and progression of NO-CAD are still not fully understood, however, inflammatory processes, particularly atherosclerosis and microvascular dysfunction are known to play a major role in it. Chemokine fractalkine (FKN/CX3CL1) is inherently linked to these processes. FKN/CX3CL1 functions predominantly as a chemoattractant for immune cells, facilitating their transmigration through the vessel wall and inhibiting their apoptosis. Its concentrations correlate positively with major cardiovascular risk factors. Moreover, promising preliminary results have shown that FKN/CX3CL1 receptor inhibitor (KAND567) administered in the population of patients with ST-elevation myocardial infarction (STEMI) undergoing percutaneous coronary intervention (PCI), inhibits the adverse reaction of the immune system that causes hyperinflammation. Whereas the link between FKN/CX3CL1 and NO-CAD appears evident, further studies are necessary to unveil this complex relationship. In this review, we critically overview the current data on FKN/CX3CL1 in the context of NO-CAD and present the novel clinical implications of the unique structure and function of FKN/CX3CL1 as a compound which distinctively contributes to the pathomechanism of this condition.

## 1. Introduction

Coronary artery disease (CAD) has been regarded as the most prevalent of cardiovascular disorders. It is the third leading cause of mortality worldwide, reportedly responsible for one third of deaths in individuals older than 35 years of age [1,2]. Clinically, CAD can present as an acute coronary syndrome (ACS) (e.g., unstable angina or acute myocardial infarction (AMI) or as a chronic syndrome with more stable symptoms (e.g., angina pectoris). Mechanistically, CAD has traditionally been associated with a flow-limiting stenosis in an epicardial coronary artery due to obstruction caused by atherosclerotic plaque.

Multiple scientific reports indicate that non-obstructive coronary artery disease (NO-CAD), defined as coronary stenosis < 50%, is increasingly recognized as an emerging clinical entity requiring further investigation [3]. Vasomotor abnormalities, coronary microvascular dysfunction and inflammation are important mechanisms underlying the occurrence of angina pectoris with NO-CAD. There is still very little available research relative to its clinical presentation, which includes: angina with non-obstructive coronary arteries (ANOCA), ischemia with non-obstructive coronary arteries (INOCA), and myocardial infarction with non-obstructive coronary arteries (MINOCA) [4]. The importance of this issue lies not only in the potential of NO-CAD to reduce quality of life [5] and consume enormous health-related resources [6], but also in its ability to increase the risk of major adverse cardiovascular events (MACE) and all-cause mortality [7,8].

Prompted by previous research assessing the role of chemokines in cardiovascular diseases, in this review we analyze a possible role of FKN/CX3CL1 in the pathological processes leading to NO-CAD. In particular, we focus on its role in the processes of inflammation, coronary microvascular dysfunction and atherosclerosis in order to elucidate the influence of FKN/CX3CL1 on vascular endothelial function. This review comprehensively summarizes these findings and their implications for a better understanding of the NO-CAD pathogenesis.

## 2. Non-Obstructive Coronary Artery Disease

Non-obstructive coronary artery disease (NO-CAD) is characterized by coronary arteries that are narrowed by less than 50%, with FFR of ≤0.80 as observed in coronary angiography. NO-CAD syndromes are increasingly recognized as heterogeneous clinical entities. The distinction between ANOCA, INOCA and MINOCA is based on the symptoms, presence of ischemia and troponin elevations. ANOCA can be diagnosed if the sternocardial pain occurs without presence of myocardial ischemia, INOCA requires appearance of ischemia and MINOCA diagnosis can be made when troponin levels are characteristically elevated as in other types of myocardial infarction (MI).

### 2.1. Prevalence of NO-CAD

According to the research, even 40% of patients that undergo coronary angiography do not present significant obstruction in coronary arteries [1]. Results of two separate studies have shown that among patients with non-ST-elevation acute coronary syndrome (NSTE-ACS), almost 10% had non-obstructive arteries [9,10]. For ST-elevation acute coronary syndrome (STE-ACS), the prevalence ranges from 10 to 25% for women and 6 to 10% for men [11]. Interestingly, based on a worldwide research, NO-CAD more commonly occurs in women than men [9,10,11,12,13]. Shaw et al. indicated that almost two-thirds of females who undergo coronary angiography have no significant obstruction in coronary arteries. These women are more likely younger (before menopause), obese, post-hysterectomy or with polycystic ovary syndrome than patients with obstructive CAD [6]. There is only limited data available regarding the specific types of NO-CAD. In a retrospective study by Patel et al., researchers have shown that approximately 24% to 26% of all patients with CAD were diagnosed with ANOCA and the percentage was higher in patients with stable angina than ACS [14]. Another study indicates that of all the patients who underwent coronary angiography, 47% had INOCA and, similarly, the percentage of women was higher than that of men [5]. The prevalence of MINOCA varies between 1 and 15% in patients with AMI [15]. According to ORPKI Polish National Registry in both STEMI and non-ST-elevation myocardial infarction (NSTEMI) patients in Poland, MINOCA occurs in 7.8% of cases [16]. In line with this, an ACTION Registry–GWTG study revealed that the prevalence of MINOCA in patients with MI was 5.9%. Moreover, MINOCA was more prevalent in women than in men (10.5% versus 3.4%) which is in accordance with the aforementioned studies [17].

### 2.2. Pathophysiology of NO-CAD

**Figure 1:**
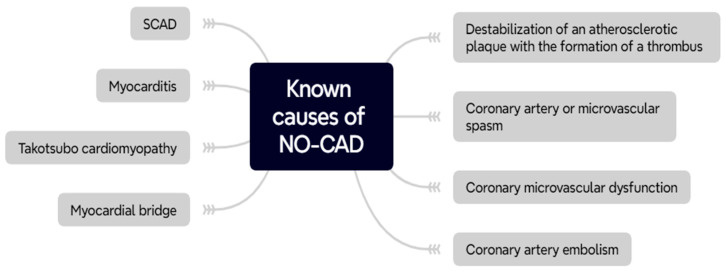
Known pathophysiological mechanisms underlying the development of NO-CAD.

NO-CAD can be caused by a variety of causes, each associated with a specific underlying pathophysiological mechanism (Figure 1). Among the most relevant, directly related to coronary vascularization are: destabilization (rupture/dissection) of atherosclerotic plaque, coronary microcirculation dysfunction (CMD), coronary artery spasm, embolism in coronary arteries and spontaneous coronary artery dissection (SCAD). Other cases of NO-CAD may be related to myocardial abnormalities such as myocarditis (parvovirus B19 infection can mimic MI similar to MINOCA [18]), Takotsubo cardiomyopathy or presence of myocardial bridges over coronary arteries. However, in some cases the mechanisms of NO-CAD overlap or the cause remains unknown. A study by Ford at al. indicated that INOCA is mainly caused by microvascular angina (52%), vasospastic angina (17%) or a mix of both (20%) [5]. According to the available research, epicardial vasospasm accounts for even up to 95% of MINOCA cases, while CMD for about 20%. Disturbances in oxygen demand and supply can also lead to MINOCA, which explains why about half of the patients with type 2 MI do not have significant obstruction [19].

#### 2.2.1. Coronary Microvascular Dysfunction

The coronary microcirculation (CM) plays a crucial role in regulating coronary blood flow to meet the dynamic needs of the myocardium. CM refers to the epicardial pre-arterioles, intramyocardial arterioles, and capillaries. The epicardial pre-arterioles (500–100 μm) not only account for most of the resistance but also respond to flow-related stimuli. Intramyocardial arterioles (<100 μm) have the highest resistance and are responsible for the metabolic regulation of coronary blood flow (CBF) in response to myocardial oxygen demand. Capillaries (<10 μm) act as exchanging vessels, due to their large surface and relatively high permeability [20]. CMD can be defined as endothelial dysfunction, vascular smooth muscle dysfunction, or both, occurring simultaneously and resulting in impaired maintenance of vascular tone [21].

Endothelial cell dysfunction (ECD) constitutes a major component of CMD. ECD leads to either impaired vasodilation or increased vasoconstriction which dysregulates the blood supply to the myocardium. Depending on the presence of pathological processes in the endothelial cells (ECs), the causes of CMD are classified as endothelium-dependent or endothelium-independent. Endothelium-dependent CMD results from the inability of ECs to produce nitric oxide (NO). Therefore, smooth muscle cells (SMCs) are unable to relax, and the coronary artery is unable to dilate as required. This results in reduced blood flow to the myocardium during times of stress, leading to angina. Endothelium-independent CMD refers to the inability of SMCs to relax despite adequate levels of NO and has similar effects to endothelium-dependent CMD. Damaged endothelium can be characterized by inflammation, changes in permeability or response to vasoactive stimuli [22].

The main molecular level of EC dysfunction is related to the vasodilatory capacity of NO. In healthy endothelium, NO is synthesized from oxygen and L-arginine in a reaction catalyzed by nitric oxide synthase (eNOS) [23]. In patients with diabetes mellitus (DM), hypertension, obesity or other diseases where chronic inflammation is present, activity of eNOS is reduced thereby leading to lower NO production and deteriorated vasodilation of coronary arteries. Reactive oxygen species (ROS) are also responsible for reducing NO levels by reacting with NO to form peroxynitrites, which are highly prooxidant and have no vasoactive function [24]. ROS can be excessively accumulated in ECs due to tumor necrosis factor-alpha (TNF-α) mediated inflammation [25].

Adhesive molecules expression, such as vascular cell adhesion molecule-1 (VCAM-1) or intercellular adhesion molecule-1 (ICAM-1), is inhibited by NO, which results in increased recruitment of immune cells and fibrotic changes in the vascular walls and myocardium [26,27]. Expression of ICAM-1, VCAM-1 and other adhesive molecules like E-selectin is induced by shear stress [28], which can appear in microvessels.

Elevated angiotensin II (Ang-II) level leads to increased activity of arginase, which reduces L-arginine available for eNOS to synthesize NO. Moreover, Ang-II mediates the release of tumor necrosis factor-beta (TNF-β) which is a pro-fibrotic cytokine [29,30]. This mechanism could partially explain elevated risk of CAD development in hypertensive patients [31].

Inflammatory states may induce endothelial production of endothelin-1 (ET-1) [32], which has a further pro-inflammatory and vasoconstricting activity when binding to ET_A_ receptors and, conversely, by binding to ET_B_ receptors. By activation, ET-1 stimulates the release of NO and prostanoids [33]. However, NO reciprocally inhibits the release of ET-1. Disruption of the balance between NO and ET-1 activity can significantly contribute to ECD [34].

The assessment of CM involves the measurement of a coronary flow reserve (CFR) and an index of microvascular resistance (IMR). CRF is determined by measuring coronary or myocardial blood flow at rest (basal flow) and with maximal hyperemia, after an intracoronary or intravenous infusion of adenosine, which mimics stress conditions, when the blood flow is maximized. CFR can be reduced both in a non-obstructive as well as in an obstructive CAD. IMR is calculated as the distal coronary pressure divided by the inverse of the mean transit time during maximal hyperemia. For simultaneous measurement of coronary pressure and hyperemic flow, this procedure requires the use of a combined pressure-temperature sensor-tipped coronary guidewire. In patients with stable non-obstructive atherosclerosis, abnormal IMR was associated with worse coronary vascular disease (CVD) outcomes [35,36]. CFR < 2.5 and IMR > 25 can be present in CMD especially in endothelial independent CMD [37].

CMD has been reported in many systemic and cardiologic diseases. The research reveals that patients with hypercholesterolaemia may have a reduced CFR, even if their arteries are normal on angiography [38]. Short-term but intensive cholesterol lowering is reported to improve myocardial perfusion [39,40]. Both type 1 and type 2 DM impair CM and reduce CFR independently of obesity, dyslipidemia or hypertension [41,42]. Myocardial ischemia in patients with arterial hypertension can also occur due to CMD despite the absence of obstructions in arteries or without left ventricle hypertrophy [43,44]. The hypertrophy of left ventricle can occur as a result of aortic stenosis or as a part of hypertrophic cardiomyopathy. Irrespective of the cause, hypertrophy in myocardial ischaemia can manifest itself in mechanisms of microvascular remodeling leading to CMD [45,46,47,48]. Smoking leads to endothelial dysfunction in central and peripheral vessels, being a well-established risk factor for cardiovascular diseases. Reduced CFR has been found in smokers, but short-term administration of antioxidants, such as vitamin C, improved blood flow in the microcirculation, suggesting the involvement of ROS in the generation of CMD [49].

#### 2.2.2. Atherosclerosis and Plaque Destabilization

The process of atherosclerotic plaque deposition is common to all CAD types. However, in NO-CAD, the narrowing of a coronary vessel is less than 50%. It is noteworthy that atherosclerosis plays a mechanistic role in most cases of non-obstructive plaques with positively remodeled epicardial coronary arteries, as demonstrated by intravascular invasive imaging diagnostic techniques, such as intravascular ultrasound (IVUS) [50], optical coherence tomography (OCT) and multi-slice coronary computed tomography angiography (CTA). The possible involvement of atherosclerosis risk factors such as diabetes, hypertension, hyperlipidaemia and smoking in the pathomechanisms of NO-CAD warrants further investigation. They may play a role both in atherosclerosis and in the microvascular dysfunction and increased vasoconstriction that underlie NO-CAD.

Inflammation and monocyte/macrophage accumulation play a major role at each stage of the atherosclerosis process. Moreover, increased number of adhesive molecules like ICAM-1 and VCAM-1 together with altered expression of chemoattractants and the loss of the endothelial barrier promote leukocytes to enter the interior of the vessel wall [51,52,53]. This applies to epicardial coronary vessels and microvessels. Inflammation occurs conjointly with the accumulation of lipids including oxidized ones by an influence of ROS. Oxidized lipids further stimulate inflammation [54]. Macrophages penetrating to intima absorb those lipids and transform to foam cells. Macrophage colony-stimulating factor (M-CSF) produced by activated macrophages recruit other monocytes, which could become foam cells in the future [55]. T-lymphocytes secrete cytokines such as interferon-γ (IFN-γ) and TNF–β, which can stimulate macrophages, ECs, and SMCs promoting their further migration and proliferation in intima. As inflammation continues, activated leukocytes release fibrotic factors as well as growth factors that also promote the replication of SMCs and fibroblasts and lead to excessive production of extracellular matrix [56]. Another important mechanism in building atherosclerotic plaque is neovascularization and angiogenesis. Local oxygen diffusion in the plaque is inefficient and leads to hypoxia, which, together with inflammation, promotes the release of angiogenic factors such as vascular endothelial growth factor (VEGF), which stimulate sprouting angiogenesis from vasa vasorum—the vessels that nourish the arteries. The newly formed vascular network facilitates leukocyte entry into atherosclerotic plaque [57]. Atherosclerotic plaque is directly covered by a fibrous cap, which can break leading to plaque rupture, and, possible occlusion of the vessel, with the resultant ACS. Proteolytic enzymes that are released by macrophages degrade collagen that strengthens the fibrous cap. IFN-γ produced by T-lymphocytes also decreases the synthesis of collagen by SMCs. These mechanisms render a thin, weak cap which can easily rupture [58]. Another example of plaque destabilization is when the plaque does not rupture completely, but only partially dissects, allowing blood flow inside the artery wall and creating the false lumen and hematoma inside. This pathomechanism is called SCAD. Intramural hemorrhage can also appear due to damage of vasa vasorum. In both situations, enlargement of the hematoma may lead to occlusion of the true lumen of the coronary artery and ACS [59,60]. SCAD tends to be more common in pregnant women and in patients with connective tissue disorders [59].

The imbalance between prothrombotic and antithrombotic activity in the atherosclerotic plaque increases with the progression of the inflammatory state in which macrophages produce excessive amounts of tissue factor (TF). ECs express more von Willebrand factor (vWF) or plasminogen activator inhibitor-1 (PAI-1), which inhibits fibrinolysis. Activated SMCs can also produce TF. In this state, a clot can form and close the vessel [61].

#### 2.2.3. Inflammation

One of the key factors responsible for atherosclerosis is inflammation, which contributes to nearly all types of CAD. Recent studies further highlight the critical role of inflammation not only in atherosclerotic processes but also in non-atherosclerotic mechanisms underlying NO-CAD. Interestingly, inflammatory response is not limited to the ruptured plaque and the adjacent myocardium, but may affect the entire heart, leading to severe consequences, such as cardiomyocyte death. Moreover, spontaneous thrombolysis, a recognized factor in NO-CAD, may be responsible for ischemia/reperfusion injury, resulting in: an increase in ROS, exacerbation of inflammation and even cell death via mechanisms such as necrosis, apoptosis, or ferroptosis [62]. A recent clinical trial confirmed this thesis, wherein the concentration of high sensitivity C-reactive protein (hsCRP), an inflammatory marker, emerged as a stronger predictor of future cardiovascular events and mortality than cholesterol concentration [63]. A higher presence of pro-inflammatory conditions in MINOCA has been described, which points to the correlation between the immune system and non-obstructive MI [64]. In the study conducted by Hjort et al., the levels of pro-inflammatory markers were compared between healthy patients, MI patients with obstructive CAD and patients with MINOCA. It is also noteworthy that three months post-MI, patients who suffered from MINOCA had significantly higher activity of inflammation biomarkers [65].

Among various inflammatory factors that activate endothelium and lead to ECD, TNF-α, interleukins, IFN-γ, binding of pathogen-associated molecular patterns (PAMPs) and damage-associated molecular patterns (DAMPs) are most prominent [66,67]. These proinflammatory compounds activate intracellular effectors such as nod-like receptor family pyrin domain-containing 3 (NLRP3) [68] or nuclear factor kappa-light-chain-enhancer of activated B cells (NF-κB) [69], which stimulate the expression of adhesive molecules (VCAM-1, ICAM-1, selectins) [51], chemoattractants—monocyte chemoattractant protein-1 (MCP-1) [52], the release of other inflammatory cytokines (IL-1β,IL-18) [68], promote pro-thrombotic activity [70,71,72] or provide loss of endothelial barrier function [53]. Adhesive molecules promote rolling, arrest and diapedesis of inflammatory cells including monocytes which are responsible for atherogenesis [51]. Enhanced permeability allows leukocytes to enter the vascular wall [53]. Released IL-1β and IL-18 further drive the inflammatory process and oxidative stress [68]. The imbalance between pro- and anti-thrombotic activity is expressed by the upregulation of TF, vWF and downregulation of thrombomodulin, leading to easier clot formation [70,71,72].

## 3. An Overview of Fractalkine

The chemokine fractalkine (FKN/CX3CL1), the only member of the CX3C subfamily, was discovered in 1987 by Bazan et al. [73]. FKN/CX3CL1 is expressed in activated or stressed endothelium, SMCs, skeletal muscle cells, macrophages, neurons, hepatocytes and adipocytes. It binds to the G-protein-coupled receptor CX3CR1, serving as its exclusive ligand. CX3CR1 is expressed on leukocytes: monocytes, macrophages, T CD4 cells, T CD8 cells, γδ T cells, dendritic cells, natural killer (NK) cells, platelets, SMCs and cardiomyocytes [74,75,76]. FKN/CX3CL1 has an unusual dual function. It appears in two forms, as a membrane-anchored molecule displaying adhesion properties and a cleaved soluble form with chemotactic properties [73]. Transmembrane FKN/CX3CL1 (tFKN/CX3CL1), expressed by inflamed vascular tissue, binds to CX3CR1 receptors on leukocytes from the bloodstream. This high-affinity binding allows leukocytes to move across the endothelium without engaging in the selectin- and integrin-mediated rolling phase required for classical leukocyte transmigration, which accelerates the process [77]. Inflammatory cytokines such as TNF-α, interleukin-1 (IL-1), and IFN-γ enhance expression of tFKN/CX3CL1 on ECs [78]. Soluble FKN/CX3CL1 (sFKN/CX3CL1) is a part of tFKN/CX3CL1 that has been cleaved from the cell surface by a disintegrin-like metalloproteinase 10 (ADAM10) or TNF-α converting enzyme (TACE) [79]. This modification allows FKN/CX3CL1 to act as a chemoattractant for immune cells [80]. In an inflammatory environment, TACE stimulated by lipopolysaccharides or interleukin-1β (IL-1β) significantly enhances the cleavage process, whereas ADAM10 predominantly oversees this process under homeostatic conditions [79]. Concurrently, the expression of FKN/CX3CL1 can be upregulated by inflammatory cytokines such as TNF-α and IL-1, through stimulation of the cluster of differentiation 40 (CD40), or even by FKN/CX3CL1 itself. Conversely, cytokines associated with T helper 2 cells, like interleukin-4 (IL-4) and interleukin-13 (IL-13), can mitigate the induction of fractalkine by TNF-α and IFN-γ, demonstrating a complex interplay of factors influencing FKN/CX3CL1 dynamics in inflammatory responses [81,82]. The intracellular effects of CX3CR1 activation are not well understood. Current findings suggest that its activation leads to stimulation of angiogenesis through the Raf-1/MEK/ERK, PI3K/Akt/eNOS pathways [83] and activation of several inflammatory signaling pathways including JAK-STAT, Toll-like receptor, MAPK, NF-κB and Wnt/β-catenin, among many others [84]. These pathways are known to inhibit apoptosis via the induced expression of anti-apoptotic molecules, such as B-cell lymphoma 2 (BCL-2), B-cell lymphoma-extra-large (BCL-xL), a critical mechanism for immune cells during inflammation. Additionally, the CX3CL1/CX3CR1 pathway leads to the formation of a positive feedback loop (increased FKN/CX3CL1 expression) via the PI 3-kinase/PDK1/Akt/NIK/IKK/NF-κB signaling pathway [81].

## 4. The Link between Fractalkine and NO-CAD

The role of the FKN/CX3CL1 in CAD is well-documented and thoroughly discussed elsewhere [85,86]. However, its association with NO-CAD requires further elucidation. The pathogenesis of NO-CAD includes structural and functional mechanisms. Structural mechanisms include perivascular inflammation, arteriolar remodeling and fibrosis, capillary rarefaction or obliteration, microthrombi and hemostatic factors. Functional mechanisms can be divided into endothelium-dependent and -independent. Atherosclerosis likewise may accompany these processes. FKN/CX3CL1 seems to be involved in those pathomechanisms given its unusual structure and function (Table 1).

### 4.1. Fractalkine—A Key Player Which Promotes Inflammation

Among the different classes of chemokines, FKN/CX3CL1 has been shown to play a major role in inflammatory diseases due to its functional and structural characteristics. In the Chronic Renal Insufficiency Cohort (CRIC) study, baseline FKN/CX3CL1 levels were associated positively with all-cause mortality and cardiovascular risk factors, such as diabetes, hypertension, hyperlipidemia, higher body mass index (BMI), and higher plasma levels of the inflammatory markers (IL-6, TNF-α, and hsCRP) in adults with chronic kidney disease [87]. Other studies confirm this correlation [113]. In line with these findings, a strong link has been found between elevated FKN/CX3CL1 levels and an increased inflammatory state, which is critical in the pathophysiology of CAD. It is worth noting that FKN/CX3CL1 is a component of the senescence-associated secretory phenotype (SASP), which further explores its role in inflammation. Cellular senescence can induce the production of various chemokines, inflammatory cytokines, extracellular matrix remodeling factors, and growth factors by SASP. These cells are implicated in the acceleration of atherosclerosis, potentially through FKN/CX3CL1 release. As such, they constitute a significant source of FKN/CX3CL1, contributing to its role in the inflammatory processes associated with cardiovascular disease progression [114,115]. Additionally, perivascular adipose tissue, under inflammatory conditions, actively contributes to the release of FKN/CX3CL1 [89]. These findings highlight the diverse cellular sources capable of releasing FKN/CX3CL1, extending beyond the conventional cell types typically associated with its secretion.

Fractalkine, as a chemokine, also acts beyond inflammatory cell recruitment. It is an adhesion molecule, which facilitates leukocyte transmigration and accelerates ECs and SMCs migration and proliferation. As previously mentioned, FKN/CX3CL1 functions as a mediator of tissue remodeling and angiogenesis caused by inflammation. Increased levels of FKN/CX3CL1 contribute to excessive monocyte and platelet activation, accelerated plaque development and increased risk of plaque rupture [77].

**Figure 2:**
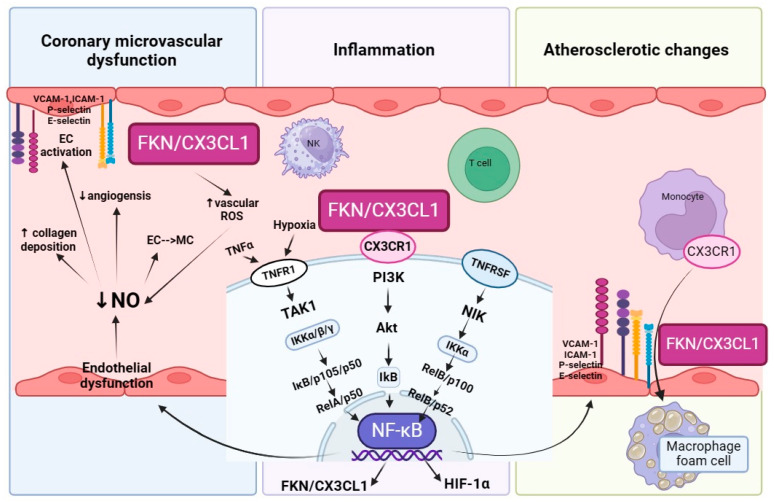
Fractalkine role in the pathomechanisms of NO-CAD. Main leading causes of NO-CAD are coronary microvascular dysfunction (CMD), atherosclerotic changes within the coronary microcirculation and inflammation, which underlies these processes. In an inflamed endothelium there is an increased production of FKN/CX3CL1, which activates multiple intracellular signaling pathways via its receptor CX3CR1. FKN/CX3CL1 can bind to CX3CR1 expressed by monocytes, T lymphocytes and natural killer cells acting as a specific adhesion molecule, attracting these cells to the artery wall inflammatory process [80]. Monocyte adhesion to the endothelium is an initial stage of atherosclerosis. Recruited monocytes then transform into macrophages, engulf lipids, and exhibit morphological features of foam cells [100]. CX3CL1/CX3CR1 interactions regulate cell migration and proliferation through the Pl3K/Act signaling pathway. FKN/CX3CL1 induces vascular dysfunction by stimulating vascular ROS resulting in reduced NO bioavailability [110]. The decreased production of NO by impaired ECs increases collagen deposition, reduces angiogenesis and promotes the conversion of ECs into mesenchymal cells, leading to microvascular rarefaction [93]. Endothelial activation—defined by endothelial expression of VCAM-1, ICAM-1, P-selectin, E-selectin—is mainly due to activation of the NF-κB signaling cascade. NFĸB controls expression of FKN/CX3CL1 [112]. NFĸB can be activated through classical complex (IKKα/β/γ) and alternative pathways (NIK-mediated activation of IKKα). In the classical pathway, NFĸB dimers RelA/p50 are released from NFĸB inhibitors (IĸB). The non-classical pathway is activated by factors of TNF receptor (TNFR) superfamily members (TNFRSF). Direct activation of NFĸB-inducing kinase (NIK) phosphorylates an IKKα, which in turn leads to the liberation of the RelB/p52 complex [116]. In hypoxia, FKN/CX3CL1, which causes the adhesion of CX3CR1 expressing immune cells to ECs, leads to EC dysfunction and the initiation of a perivascular inflammatory response. Hypoxia and TNF-α increase hypoxia-inducible factor-1α (HIF-1α) expression via activation of the NFĸB classical pathway [117]. The figure was created in BioRender.com, accessed on 27 March 2024.

The perivascular inflammation can act as a driving force in the development of subsequent medial and intimal remodeling, which underlies CMD [93,109]. FKN/CX3CL1 and other chemokines have been shown to participate at every step of the remodeling process [90]. Moreover, based on the available research, perivascular fibrosis was less prominent in mice treated with the FKN/CX3CL1 neutralizing antibody [91]. FKN/CX3CL1 was reported to promote fibrogenesis [92] and induce vascular dysfunction by stimulating vascular ROS resulting in reduced NO bioavailability [110]. The decreased production of NO by impaired ECs increases collagen deposition, reduces angiogenesis and collateral development. It also promotes the conversion of ECs into mesenchymal cells, resulting in a loss of perfused microvessels, fibrosis and hypertrophy, known collectively as microvascular rarefaction [94,95] (Figure 2).

### 4.2. Fractalkine Underlies the Onset of Atherogenesis and Further Enhances This State

Inflammatory activation of the vascular endothelium is a key factor in the development of atherosclerosis [118]. Monocyte infiltration of the vessel wall is an early and essential step in atherogenesis [119]. Interestingly, FKN/CX3CL1—known to be an endothelial-associated rather than a soluble cytokine—was shown to predominate in early atherosclerotic lesions. The research revealed that FKN/CX3CL1 can locally enhance the adhesion of monocytes and T cells, whereas at later stages soluble chemokines for a variety of leukocytes were detected. Moreover, the scientists reported that the endothelial glycocalyx-binding interferon γ-induced protein 10 (IP-10) was expressed specifically in lesional endothelium, but its corresponding protein was also detected in artery sections without plaques, which was consistent with locally enhanced plasma levels [101]. On the contrary, FKN/CX3CL1 is anchored in the endothelium by a trans-membrane domain, a protein module that is expressed specifically in ECs [78]. Therefore, its protein was detected much more discriminatively in endothelium overlying early lesions only [103]. In line with this, another study revealed that FKN/CX3CL1 was substantially expressed at very early stages of atherosclerosis, which further points to its key role in the initial stages of lesion development, in addition to its relevance in advanced disease [102,104]. Hence, if being considered as biomarker, FKN/CX3CL1, holds a great potential to detect early atherosclerosis (Table 1).

The association between FKN/CX3CL1 and the infiltration of the vessel wall by immune cells has also been supported by recent studies in mice, where deletion of the CX3CR1 gene (CX3CR1^−/−^ mice) or use of a CX3CR1 inhibitor led to a significant reduction in macrophage infiltration and, consequently, less advanced atherosclerosis [102,105,120]. Of note, the macrophages are a source of FKN/CX3CL1 in atherosclerotic plaque [86,121,122]. CXCR1 activation also resulted in platelet degranulation. The released factors not only promoted monocyte transmigration but also facilitated platelet aggregation, both of which are critical for the progression of atherosclerotic plaques [96,123]. Another study in mice revealed that the inhibition of CXCR1 prior to reperfusion resulted in reduced infarct size by 50% [124].

While comparing patients with stable and unstable angina, CX3CR1 expression and FKN/CX3CL1 concentration was higher in the latter group [125]. Similarly, FKN/CX3CL1 levels were more elevated in patients with AMI after PCI than in patients with stable angina, but the values equalized after 24 h in both groups. No correlation was found between FKN/CX3CL1 levels and infarct size [126]. Another independent study revealed that the level of sFKN/CX3CL1 was positively associated with N-terminal pro-B-type natriuretic peptide (NT-proBNP) 30 days after PCI [127]. It was also noted that FKN/CX3CL1 contributed to lymphocyte transmigration in STEMI patients. Moreover, post-PCI lymphopenia associated with these processes was positively correlated with poor diagnosis due to increased microvascular obstruction [128]. In addition to its role in leukocyte migration, FKN/CX3CL1 also affects angiogenesis, which is a crucial process in atherosclerotic plaque growth, destabilization and rupture [57].

### 4.3. Fractalkine Promotes Coronary Microvascular Dysfunction

Coronary microvascular dysfunction is among the main leading causes of NO-CAD. Endothelial dysfunction and vascular smooth muscle cell hyperreactivity may lead to impaired vascular tone, vasodilatation and/or enhanced vasoconstriction [129]. FKN/CX3CL1—induced by primary proinflammatory signals in vascular endothelial cells—is substantially involved in the pathogenesis of vascular dysfunction. It promotes the production of ICAM-1 via the CX3CR1/PI3K/Akt/NF-κB pathway [111], which is elevated in CMD [112]. FKN/CX3CL1 promotes vascular dysfunction through the stimulation of vascular ROS, consequently leading to a decrease in NO bioavailability [110] (Figure 2). Moreover, FKN/CX3CL1-mediated activation and adherence of NK cells can inflict endothelial cell damage, a process that occurs even in the presence of autologous major histocompatibility complex [130]. The interaction between FKN/CX3CL1 and vascular dynamics is further complicated by the influence of shear stress. Elevated laminar shear stress—defined as the force per unit area exerted by blood flow on the endothelial lining of blood vessels—reduces the expression of endothelial FKN/CX3CL1 at both the mRNA and protein levels, and also diminishes its induction by TNF-α. This type of shear stress lessens the adhesiveness of ECs to CX3CR1-positive monocytes, thereby opposing vascular inflammation [107]. Accordingly, low shear stress upregulates FKN/CX3CL1 expression [106] via the mitogen-activated protein kinase (MAPK) pathway [108]. It is noteworthy that low coronary wall shear stress is also linked with pronounced endothelial dysfunction in patients with NO-CAD [131,132]. In addition to these roles, FKN/CX3CL1 is also involved in the proliferation of vascular smooth muscle cells (VSMCs). Studies have shown that CX3CR1−/− mice exhibit a lower incidence and slower progression of intimal hyperplasia following vascular injury. This indicates that the presence or absence of FKN/CX3CL1, and its receptor, can significantly influence the development of structural changes within the vessel wall [133].

### 4.4. Future Implications for Fractalkine Role in NO-CAD

Several attempts have already been made to block the FKN/CX3CL1 axis in cardiovascular diseases and others. Pharmacological inhibition of CX3CR1 in murine models of atherogenesis led to reduced atherosclerotic lesion development and, interestingly, attenuated monocytosis. It was showed that the antiatherogenic potency of CX3CR1 blockade was related to reduced CX3CR1-dependent adhesion and survival of inflammatory monocytes. The researchers concluded that controlling monocytosis by blocking CX3CR1 may help prevent atherogenesis [105]. Despite CX3CR1 being identified as a highly attractive target for therapeutic interventions, to date, KAND567 is the only agent targeting the CX3CR1. It is a selective, non-competitive, allosteric antagonist of the CX3CR1. As of now, it is the only agent targeting the CX3CR1. Its efficacy has been demonstrated in vitro and in rodent models [134]. Currently, KAND567 is undergoing clinical trials in patients with STEMI undergoing PCI. The top-line results from this exploratory phase IIa study have recently been published, indicating that KAND567 not only proved to be safe and tolerable in patients but also demonstrated effectiveness as a treatment, with indications of clinically relevant cardioprotective effects. Notably, the study showed a reduction in the incidence of intramyocardial hemorrhage, with 38% in the KAND567 group compared to 57% in the placebo group. Additionally, there was a reduction in the incidence of left ventricular thrombosis, observed at 3% in the KAND567 group versus 15% in the placebo group. These early results indicate that KAND567 may be an effective therapeutic agent for the protection against reperfusion injury. Furthermore, in a novel study KAND567 eluting stents exhibited ~60% reduced in-stent stenosis compared to bare metal and polymer-only coated stents 4 weeks after deployment. Moreover, there was a notable decrease in peri-stent inflammation, while reendothelialization remained unaffected [134].

An interesting research strategy would involve assessing the role of FKN/CX3CL1 in NO-CAD clinical presentations and in patients diagnosed with obstructive CAD. The potential for early stratification of patients prone to develop such conditions based on FKN/CX3CL1 concentration, with a further possibility to inhibit it requires undertaking thorough research. The preliminary results of the hitherto undertaken attempts are very promising.

## 5. Conclusions

Altogether, the available data suggest that FKN/CX3CL1 is involved in the pathogenesis of NO-CAD. Considering that this condition is still not fully understood, and its occurrence is associated with high risk of cardiovascular events, any attempt to understand the causes of NO-CAD is of great clinical relevance. There is solid scientific evidence that FKN/CX3CL1 is lower under physiological conditions, whereas its increased expression is initiated by vascular dysfunction, early atherosclerotic pathomechanisms in the CM, the presence of proinflammatory cytokines and upregulated via activation of the NF-κB pathway. Therefore, blocking the CX3CL1/CX3CR1 axis may lead to a reduction in NO-CAD progression and the associated risk of MACE, which warrants further in-depth evaluation.

## Figures and Tables

**Table 1:** Fractalkine role in the processes with the potential of being identified in NO-CAD.

Processes	References
Inflammation	[80,81,87,88,89]
Arteriolar remodeling and fibrosis	[90,91,92]
Capillary rarefaction or obliteration	[93,94,95]
Microthrombi (platelet activation) and hemostatic factors	[96,97,98]
Low shear stress/Atherogensis/Atherosclerosis	[77,80,99,100,101,102,103,104,105,106,107,108]
Endothelial/Vascular dysfunction	[90,91,92,93,94,109,110,111,112]

## Abbreviations

ACS, acute coronary syndrome; ADAM10, a disintegrin-like metalloproteinase 10; AMI, acute myocardial infarction; ANOCA, angina with non-obstructive coronary arteries; Ang-II angiotensin II; BCL-2, B-cell lymphoma 2; BCL-xL, B-cell lymphoma-extra-large; BMI, body mass index; CAD, coronary artery disease; CBF, coronary blood flow; CCR2, C-C motif chemokine receptor 2; CFR, coronary flow reserve; CD40, cluster of differentiation 40; CM, coronary microcirculation; CMD, coronary microcirculation dysfunction; coronary vascular disease, CVD; DAMPs, damage-associated molecular patterns; DM, diabetes mellitus; ECs endothelial cells; ECD, endothelial cell dysfunction; ET-1, endothelin-1; FKN/CX3CL1, fractalkine; FFR, fractional flow reserve; hsCRP, high sensitivity C-reactive protein; HIF-1α, hypoxia-inducible factor-1α; IMR, index of microvascular resistance; ICAM-1, intercellular adhesion molecule-1; IFN-γ, interferon-γ; interferon γ -induced protein 10 (IP-10 IVUS, intravascular ultrasound; INOCA, ischemia with non-obstructive coronary arteries; IL-1, interleukin-1; IL-1β, interleukin-1β; IL-4, interleukin-4; IL-6, interleukin-6; IL-13, interleukin-13; MACE, major adverse cardiovascular events; M-CSF, macrophage colony-stimulating factor; MAPK, mitogen-activated protein kinase; MINOCA, myocardial infarction with non-obstructive coronary arteries; MI, myocardial infarction; MCP-1, monocyte chemoattractant protein-1; CTA, multi-slice coronary computed tomography angiography; NO, nitric oxide; eNOS, nitric oxide synthase; NLRP3, nod-like receptor family pyrin domain-containing 3; NO-CAD, non-obstructive coronary artery disease; NSTE-ACS, non-ST-elevation acute coronary syndrome; NSTEMI, non-ST-elevation myocardial infarction; NT-proBNP, N-terminal pro-B-type natriuretic peptide; NF-κB, nuclear factor kappa-light-chain-enhancer of activated B cells; OCT, optical coherence tomography; PAMPs, pathogen-associated molecular patterns; PCI, percutaneous coronary intervention; PAI-1, plasminogen activator inhibitor-1; ROS, reactive oxygen species; SASP, senescence-associated secretory phenotype; SCAD, spontaneous coronary artery dissection; SMCs, smooth muscle cells; sFKN/CX3CL1, soluble fractalkine; STE-ACS, ST-elevation acute coronary syndrome; STEMI, ST-elevation myocardial infarction; TACE, tumor necrosis factor-alpha converting enzyme; TF, tissue factor; tumor necrosis factor-alpha; tFKN/CX3CL1, transmembrane fractalkine; TNF-α, tumor necrosis factor-alpha; TNF-β, tumor necrosis factor-beta; VSMCs, vascular smooth muscle cells; VCAM-1, vascular cell adhesion molecule 1; VEGF, vascular endothelial growth factor; vWF, von Willebrand factor.
